# Determination of persistent organic pollutants in urban and peri-urban wastewater sludge: environmental and carcinogenic human risk assessment in the case of land application

**DOI:** 10.1007/s11356-024-34420-5

**Published:** 2024-07-22

**Authors:** Maria Concetta Bruzzoniti, Vander Tumiatti, Armando Quazzo, Mihail Simion Beldean-Galea, Massimo Del Bubba, Luca Rivoira

**Affiliations:** 1 Department of Chemistry, University of Turin, Via P. Giuria 5, 10125 Turin, Italy; 2 Sea Marconi Technologies, Via Ungheria 20, 10093 Collegno, Italy; 3 Società Metropolitana Acque Torino, SMAT, S.P.A. Corso XI, Febbraio 22, 10152 Turin, Italy; 4 Faculty of Environmental Science and Engineering, Babes-Bolyai University, Cluj-Napoca, Romania; 5 Department of Chemistry “Ugo Schiff”, University of Florence, Via Della Lastruccia 3, 50019 Sesto Fiorentino, Italy

**Keywords:** Wastewater treatment sludge, Polycyclic aromatic hydrocarbons, Polychlorinated biphenyls, Microwave-assisted extraction, Environmental and carcinogenic human risk analysis

## Abstract

The management of digested sludge derived from treatment water plants is a problem worldwide due to the possible transfer of contamination from sludge to amended soil and, in turn, to humans. Within this work, through a chemometric experimental design, a robust GC–MS method for the simultaneous determination of two classes of micropollutants of concern (polycyclic aromatic hydrocarbons—PAHs—and polychlorinated biphenyls—PCBs—including dioxin-like compounds) was developed using microwave-assisted extraction (MAE). The method, which showed interesting greenness features in compliance with the 12 principles of Green Chemistry, allowed PCB and PAH extraction with recoveries higher than 75% (RSD < 14%) with method detection limits between 4.6 and 11.5 µg kg^−1^ (PAHs) and between 6.9 and 13.7 µg kg^−1^ (PCBs), without preconcentration. The matrix effect was below 20%. The validated protocol allowed the characterization of two digested sludges sampled in an urban and a peri-urban district, representative models of two scenarios of different anthropic impacts. This study highlighted higher contamination for the sludge derived from the urban area, accounted for by the 4-ring PAHs. In addition, since carcinogenic PAHs were detected, the environmental risk (by mean of the comparison of predicted vs predicted no-effect concentrations) and the carcinogenic human risk from dermal contact (through the calculation of the adsorbed lifetime average daily dose) were assessed in the case of soils amended with the sludges considered, pointing out that the measured concentrations do not pose a risk.

**Supplementary Information:**

The online version contains supplementary material available at 10.1007/s11356-024-34420-5.

## Introduction

Biological sludge is generated in wastewater treatment plants (WWTPs) during secondary treatment processes such as activated sludge, membrane bioreactors, percolating filters, and rotating biological contactors. The management of sludge represents one of the most relevant challenges for environmental policies worldwide, covering both quantitative and qualitative aspects (Herrera-Navarrete et al. 2022). In fact, according to the most recent available data (years 2017–2020, depending on the accessible datasets), the amount of sludge produced in European countries stands at around ten million tons per year (European Commission and Eurostat 2022, Istituto Superiore per la Protezione e la Ricerca Ambientale (ISPRA) (2019)), most of them disposed in agriculture (≈27%), used to produce compost (≈21%), landfilled (≈8%), or incinerated (≈23%). Thermal conversion of sludge is a disposal mode, which can inhibit its intrinsic toxicity due to potentially pathogenic microorganisms and poorly biodegradable ubiquitous organic micropollutants, such as polycyclic aromatic hydrocarbons and polychlorinated biphenyls which are regulated in sludges by national and European legislation. To the best of our knowledge, the EU proposed the limits of 6 mg kg^−1^ dry matter (DM) for some selected PAHs, as the sum of acenaphthene, fluorene, phenanthrene, fluoranthene, pyrene, benzo[b,j,k]fluoranthene, benzo[a]pyrene, indeno[1,2,3-cd]pyrene, and benzo[ghi]perylene. Furthermore, a limit of 0.8 mg kg^−1^ DM of PCBs was established for congeners 28, 52, 101, 118, 138, 153, and 180 (European Commission 2000).

PAHs and PCBs, besides being present as residual compounds in treated wastewaters (Rivoira et al. 2019), have indeed been found in sewage sludge derived from wastewater treatment (Mailler et al. 2014, 2017). The accurate monitoring of these compounds in sludge is of paramount importance to avoid the leaching of micropollutants in soil and water and possible transfer in crops (Renai et al. 2021; Song et al. 2016) grown up on soils amended with sewage sludge (Boumalek et al. 2019) and for elaborating a reliable risk analysis. Furthermore, beyond the importance of PAHs and PCBs due to their intrinsic toxicity, they can be monitored as model molecules useful for studying the partition of hydrophobic micropollutants from sludge to other environmental matrixes and vice versa.

Existing methods for the determination of PAHs and PCBs in sludge rely on two different gas chromatographic procedures on extracts obtained with Soxhlet extraction. These procedures are both time- (~ 18 h) and solvent- (~ 280 mL) consuming. Moreover, they need clean-up procedures which are different for PAHs and PCBs and require large solvent volumes (320–620 mL) (Ju et al. 2009; Stevens et al. 2003). It should also be noted that the overall procedures are not validated in sludge.

Based on these considerations, the aim of this work was the optimization of an analytical method for the simultaneous analysis of thirty compounds belonging to PAH and PCB families, which include the compounds regulated by the EU proposal (European Commission 2000), through a green approach based on microwave-assisted extraction (MAE). To our knowledge, such an approach is lacking in the current literature. The microwave extraction conditions have been derived following a chemometric optimization. Since a single protocol for the analysis of PAHs and PCBs in sewage sludge is not available in the literature, the optimized method was compared with those previously published for the analysis of individual classes of compounds, considering both the analytical figures of merit and the green characteristics according to the twelve green chemistry principles (Anastas et al. 2000; Pena-Pereira et al. 2020).

The method developed was tested on two samples of sludge, deriving from urban and peri-urban WWTPs, thus allowing the simultaneous characterization of PAH and PCB contamination in two representative scenarios of different anthropic impacts. The two sludges analyzed here derived from areas with a high number of equivalent inhabitants, and they indeed could be a source of significant quantities of sludge for agricultural reuse. Hence, the possible environmental risk and the carcinogenic risk of human exposure through dermal contact (farmers/gardeners) were further investigated in the case of soils amended with the sludges considered.

## Materials and methods

### Reagents

The solvents dichloromethane and cyclohexane utilized in this study were sourced from VWR Chemicals, located in Radnor, PA, USA. Additionally, acetone and sulfuric acid with a concentration of 95–97% were obtained from Honeywell Research Chemicals in Charlotte, NC, USA. For high-purity water, having a resistivity of 18.2 MΩ cm at 25 °C, we produced it on-site using an Elix-Milli Q Academic system from Millipore, based in Billerica, MA, USA. The compounds considered in this work were the sixteen US-EPA PAHs and the main PCBs found in monitoring campaigns. In more detail, each compound is listed, with its acronym, in Table S1 of the “Supplementary information” section, for a total of thirty organic micropollutants. PAHs and PCBs were sourced from Chemical Research 2000 in Rome, Italy.

Eight isotopically labeled compounds (Chemical Research 2000) for PAHs, each with a concentration of 5 mg L^−1^, and five for PCBs (Chemical Research 2000), at 2 mg L^−1^, as listed in Table S1, were utilized as internal standards and surrogates.

Extracts were purified with silica cartridge from Waters Italy, (SepPak 690 mg, 55–105 µm).

### Instrumentation

The extraction of PAHs and PCBs was performed using a Discover microwave synthesizer from CEM, Bergamo, Italy. This microwave is equipped with software that enables the adjustment of various parameters such as Ramp Time, temperature, pressure, microwave power, and the speed of sample stirring. Additionally, a Jouan Centrifuge from ThermoFisher, Massachusetts, USA, was employed for the centrifugation of extracts.

PAHs and PCBs were analyzed using instrumental conditions described elsewhere (Bruzzoniti et al. 2022) and reported in the “Supplementary information” section.

Single ion monitoring (SIM) mode at the proper m/z ratios (see Table S1) was used for compound quantitation.

### Sludge derived from wastewater treatment

Two sludges were chosen as model matrices representative of different urban contamination situations. In more detail, dehydrated anaerobically digested sludge samples were from local urban (domestic and industrial) wastewater treatment plants located in urban (sludge #1) and peri-urban areas (sludge #2) of the Piedmont Region (North Italy). The plant located in the urban area is much more impacted by industrial wastewater in respect to the plant located in the peri-urban area. The two plants currently treat wastewater from a population equivalent of about 2,000,000 and 70,000 equivalent inhabitants. Sludge samples were desiccated at 60 °C for 24 h, homogenized, and extracted.

### Design of experiments

A full factorial experimental design was employed to fine-tune the microwave-extraction conditions. This optimization process involved varying three factors over two levels (e.g., high and low): the weight of the sludge (0.25 g and 0.5 g), the type of extraction solvent (cyclohexane and CH_2_Cl), and the extraction temperature (50 °C and 110 °C). The layout of this experimental design, which is elaborated in the “Design of experiments” section (Results and Discussion), can be found in Table 1, see Results and Discussion section. The apparent recovery percentage (E%) was used as the response variable or dependent factor.

Before extraction, proper amounts of the nine surrogate compounds (Table S1) were spiked in the sludge sample to obtain a final concentration in the extract of 1 µg L^−1^ (*C*_s_). This procedure allows us to obtain an alternative to a conventional certified reference material with the same advantages within method validation. For each test, E% was calculated for all the surrogate PAH and PCB standards, according to the Eq. (1):

1 $$E\% =\frac{Ce }{Cs }\times 100$$

where *C*_e_ (µg L^−1^) is the surrogate concentration after extraction calculated by external standard calibration.

### Final extraction and purification conditions

The finalized extraction conditions and the subsequent clean-up process are outlined as follows: A sample of 0.5 g of sludge was measured and placed into a 50 mL Pyrex vessel along with 10 mL of cyclohexane and 1 mL of CH_3_COCH_3_ for the extraction of PAHs and PCBs. The microwave digestor settings applied were a gradual increase from 0 to 110 °C over 10 min, maintaining a pressure of 350 psi and a power of 300 W. Post-extraction, the solution underwent centrifugation at 1970 × *g* for 5 min. For clean-up, the extract was passed through a Sep-Pak Silica cartridge pre-conditioned with 10 mL of cyclohexane, which helped remove organics that might have coextracted during MAE. The eluted extract (5 mL) was then treated with 2 mL of concentrated H_2_SO_4_ for 30 min to eliminate co-extracted polar substances and any residual water. Finally, 1 mL of the extract was fortified with an internal standard solution of PAHs and PCBs to attain a concentration of 5 µg L^−1^, and this prepared sample was subsequently analyzed using GC–MS.

### Figures of merit

Limits of detection and limits of quantitation of the method (MDL and MQL, respectively) for PAHs and PCBs shown in Table S2 were evaluated according to the procedure of Shrivastava and Gupta, as detailed in paragraph S2 of the Supplementary information (Shrivastava and Gupta 2011). The intra- and inter-day precisions were calculated using 20 determinations for sludge spiked with surrogate standards, on a single day/three separate days, respectively.

The matrix effect (ME) was initially assessed by preparing two separate calibration curves for each PAH and PCB surrogate and comparing their slopes (Table S3) using a statistical *t*-test approach (Slutsky 1998). Specifically, one calibration curve was derived from standard solutions prepared in cyclohexane. For the other curve, three blank sludge samples were processed using the optimized extraction method, and the calibration points were then established in this post-extracted sludge solution, deriving a matrix-matched calibration approach. Each curve included 10 calibration levels (0.15 − 6.8 µg L^−1^), with each level replicated twice.

Furthermore, to identify any suppressive or signal-enhancing effects, the matrix effect was evaluated through Eq. (2) at three specific concentration levels (10, 30, and 80 µg kg^−1^) spiked into the extracted sample.

2 $$ME\left(\%\right)=100\times \left|\frac{{A}_{\mathrm{std},\mathrm{matrix}}-{A}_{\mathrm{std},\mathrm{solvent}}}{{A}_{\mathrm{std},\mathrm{solvent}}}\right|$$

where *A*_std,matrix_ is the chromatographic area of standards prepared in the post-extracted solution, and *A*_std,solvent_ is the chromatographic area of the same concentration of standards in cyclohexane.

### Risk assessment for sludge application

The digested sludges were additionally studied for their suitability in land application. The environmental risk and the risk of human exposure through dermal contact were assessed according to the procedures hereafter described, after the calculation of the predicted pollutant concentrations in the soil.

#### Predicted concentrations in soil

The predicted concentrations of PAHs and PCBs in soil (PEC_i_) were assessed according to the European Technical Guidance Document on Risk Assessment EUR 20418 EN/2 (European Commission Joint Research Centre 2003) as reported by Verlicchi and Zambello (2015), see Eq. 3, under the assumption that complete mixing between soil and sludge occurs:

3 $${PEC}_{i}=\frac{{C}_{\mathrm{i},\mathrm{sludge}} \bullet {APP}_{\mathrm{sludge}}}{{Depth}_{\mathrm{soil}}\bullet {RHO}_{\mathrm{soil}}}$$

where *C*_i,sludge_ is the concentration of the pollutant measured in digested sludge (µg kg^−1^ DM), *APP*_sludge_ is the application rate of the dry sludge onto the soil, generally 0.5 kg m^−2^ y^−1^ for agricultural soil, *Depth*_soil_ is the mixing depth, generally 0.20 m for agricultural soils, and *RHO*_soil_ is the bulk density of wet soil, 1700 kgm^−3^ for agricultural soils (Verlicchi & Zambello 2015).

#### Environmental risk assessment due to PAHs in sludge-amended soil

To evaluate the environmental risk posed by micropollutants after sludge’s application to the soil for agriculture purposes, for each *i-*pollutant, the risk quotient (*RQ*_i_), the ratio between pollutant concentration in the amended soil (expressed by the *PEC*_i_ after one dose of sludge application) and its predicted no-effect concentration (*PNEC*_i_) was calculated (Verlicchi & Zambello 2015):

4 $${RQ}_{\mathrm{i}}=\frac{{PEC}_{\mathrm{i}}}{{PNEC}_{\mathrm{i}}}$$

The PNEC soil values for PAHs used here were taken from Sun et al. (2019)

#### Dermal contact with sludge-amended soil; carcinogenic risk

The potential for exposure via dermal contact with sludge-amended soil was assessed considering the exposure among residential adult gardeners following EPA guidelines (United States Environmental Protection Agency (2004)):

5 $${LADD}_{\text{soil contact dermal}}=\frac{{C}_{\mathrm{soil}} \bullet CF\bullet {\frac{SA}{BW}\bullet AF}_{\mathrm{soil}}\bullet EF\bullet ED\bullet ABS}{AT}$$

where *LADD*_soil contact dermal_ is the adsorbed lifetime average daily dose from dermal contact with contaminated soil (mg kg^−1^ day^−1^); *C*_soil_ is the concentration of the contaminant in the soil, equivalent to PEC in this case (mg kg^−1^), expressed as benzo[a]pyrene concentration; *CF* is the conversion factor (10^−6^ kg mg^−1^); *SA/BW* is the surface area of the skin that contacts the soil (cm^2^ event^−1^) divided by body weight (kg); *AF*_soil_ is the adherence factor for soil (mg cm^−2^); *EF* is the exposure frequency (events year^−1^); *ED* is the exposure duration (year); *ABS* is the absorption fraction (chemical specific); *AT* is the averaging time (days).

The carcinogenic risk (*R*) was calculated according to Eq. 6:

6 $$R={LADD}_{\text{soil contact dermal}}\bullet CSF$$

where *CSF* is the cancer slope factor for benzo[a]pyrene.

The input parameters, which are derived from the EPA guidelines (United States Environmental Protection Agency (2004)), are collected in Table S4 in the “Supplementary information” section.

## Results and discussion

To address the complex nature of the sludge matrix and to ensure maximum extraction recoveries with minimal interference from matrix components, both the MAE procedure and the clean-up step preceding the chromatographic analysis were meticulously optimized.

Optimization was directly performed on the sludge sample of higher complexity derived from urban, industrialized district (sludge #1).

### Method optimization

#### Design of experiments

In the MAE approach, the parameters most affecting the extraction recovery are, typically, the type of extraction solvent and temperature. Due to the complexity of the matrix and to achieve low method quantitation limits, the amount of sludge was also selected as an additional factor.

The two extraction solvents selected were cyclohexane and CH_2_Cl_2_, according to literature data for soil analysis (Bruzzoniti et al. 2012) (Ben Salem et al. 2016). The two solvents are compatible with the subsequent gas chromatographic analysis. Cyclohexane was preferred over the commonly used hexane because of its lower impact on worker exposure according to National Institute for Occupational Safety and Health (NIOSH) recommendations (National Library of Medicine, National Library of Medicine).

The two solvents (10 mL each) were mixed with a polar solvent (1 mL of acetone) to enhance microwave energy absorption and rapid heating, as detailed elsewhere (Ingrando et al. 2022).

In this study, eight experimental tests (outlined in Table 1) were conducted on desiccated sludge samples. These samples were spiked with surrogate standards. Following the MAE step, the extracts underwent a purification process to remove interfering polar compounds without interaction with analytes (Bruzzoniti et al. 2015). For this purpose, a Sep-Pak Silica cartridge was used. Post-cleanup, the extracts were treated with H_2_SO_4_ prior to their analysis via GC–MS, as elaborated in the “Final extraction and purification conditions” section of the study.

**Table 1:** Experimental design matrix used throughout this work. Extraction recoveries are referred to PAH and PCB surrogates

Run	Factors			Extraction recovery (%)	Comments
	Sludge weight (g)	Solvent^a^	Temperature (°C)		
1	0.25	CH_2_Cl_2_	50	PCBs: n.d. – 170; PAHs: n.d. – 280	Strong matrix effect (co-extracted interferences)
2	0.25	CH_2_Cl_2_	110		
3	0.5	CH_2_Cl_2_	50		
4	0.5	CH_2_Cl_2_	110		
5	0.5	Cyclohexane	110	PAHs: 75 – 130; PCBs: 107 – 121	All PAHs extracted
6	0.5	Cyclohexane	50	PAHs: n.d. – 88; PCBs: 93 – 115	Ind, DBA not extracted
7	0.25	Cyclohexane	110	PAHs: n.d. – 84; PCBs: 125 – 144	Ind, DBA not extracted
8	0.25	Cyclohexane	50	PAHs: n.d. – 56; PCBs: 123 – 154	Only BaP extracted

*n.d.* not detected

^a^10 mL + 1 mL CH_3_COCH_3_

Despite each test including a purification step after the MAE extraction, extraction performed in CH_2_Cl_2_ (experimental tests no. 1, 2, 3, 4) showed high GC–MS background noise with respect to cyclohexane (Fig. S1), with E% within 0–170% for PCB and 0–280% for PAH surrogates, indicating a strong matrix effect due to co-extracted interferents. Extraction data are reported in Table 1. The higher extraction capabilities of CH_2_Cl_2_ than cyclohexane are ascribed to the higher ability to convert electromagnetic energy into heat (Gabriel et al. 1998; Mello et al. 2014).

**Fig. 1:**
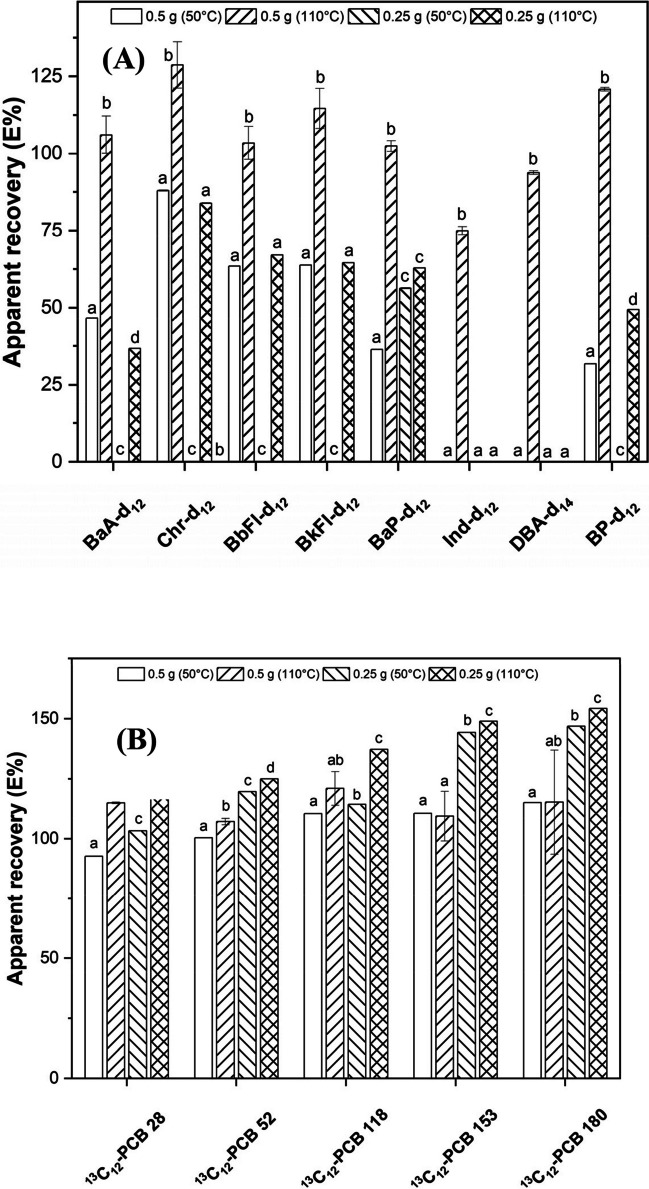
Effect of the amount of sludge and extraction temperature in MAE extraction with cyclohexane (experimental tests no. 5, 6, 7, and 8) on extraction recovery (E%) of PAHs (**A**) and PCBs (**B**). Mean values and error bars (*n* = 3) for standard deviation are also reported. Bars labeled with different letters pointed out statistically different mean values (Dunnett T3 nonparametric contrast test, *p* ≤ 0.05). Conversely, bars with at least one letter in common represent mean values that are not statistically different from each other. Instrumental conditions are reported in the manuscript

Extraction recoveries highly exceeded the quantitative extraction target of around 100%, especially for the experiments performed in dichloromethane, so, data were not further treated by conventional approaches (e.g., Pareto diagrams (Truzzi et al. 2014), Yates algorithms (Moradi 2021), and surface response plots). However, the experimental design provides interesting observations for E% values obtained using cyclohexane (runs no. 5, 6, 7, and 8) reported in Table 1 and Fig. 1A for PAHs and in Fig. 1B for PCBs. Data show that extraction of PAHs is more influenced by the studied factors than PCBs, as confirmed by the Dunnett T3 contrast test (statistical similarities, pointed out by similar letters, are more present in this latter class of target pollutants). In more detail, the sludge amount is a crucial parameter to achieve satisfactory recoveries for PAHs, especially for the highest molecular weight compounds Ind-d_12_ and DBA-d_14_, which are not extracted at all using 0.25 g of sample (tests no. 7, 8). Also, the temperature is a crucial parameter to extract PAHs since the use of the higher temperature (110 °C) improves E%, this behavior being more evident when extracting 0.5 g sludge. In addition, it should be mentioned that for runs no. 5, 6, 7, and 8, optimal reproducibility was achieved for the analytes (both PAHs and PCBs), with almost all the relative standard deviation values, RSD, (*n* = 3 replicates) far lower than 10% (generally indicative of a reliable and reproducible method in environmental monitoring (Cloutier et al. 2017).

To summarize, according to the experimental design, experiment no. 5, i.e., 0.5 g sludge extracted in cyclohexane:CH_3_COCH_3_ 10:1 (mL) at 110 °C, provided the optimized extraction conditions (recoveries ranging from 75 to 130% for PAHs and from 107 to 121% for PCBs, RSD% lower than 10%). Worth mentioning that even if PAHs are characterized by significant volatility (Castro et al. 2009), the abovementioned extraction conditions avoid loss of analytes.

### Method validation

#### Linearity, MDLs and MQLs, and precision

The linearity of the method was evaluated directly within the sludge matrix over ten calibration levels. For PAHs, the linearity range was determined to be between 0.15 and 3.5 µg L^−1^, while for PCBs, it was between 0.3 and 6.7 µg L^−1^. The coefficients of determination (*R*^2^) were found to fall between 0.974 and 0.997.

The MDLs for PAHs varied from 4.6 to 11.5 µg kg^−1^ and for PCBs, from 6.9 to 13.7 µg kg^−1^. As for the MQLs, they were found to be between 14.1 and 34.8 µg kg^−1^ for PAHs and between 20.8 and 41.6 µg kg^−1^ for PCBs. Data for each analyte is presented in Table S2. The abovementioned limits are achieved without any preconcentration step and are comparable to, or lower than those achieved by MAE extraction, solvent change, preconcentration, and analysis by liquid chromatography with fluorescence detection (Flotron et al. 2003; Villar et al. 2004). Comparisons for PCBs are less straightforward due to the lack of method performance data, except for recoveries.

The method developed within this study for the simultaneous analysis of PAHs and PCBs achieves quantitation limits that are significantly lower than the maximum concentration limits proposed by the European Commission for sludge. These limits are set at 6000 µg kg^−1^ for PAHs and 800 µg kg^−1^ for PCBs. This demonstrates the high sensitivity and effectiveness of the method in detecting even low concentrations of these compounds in sludge samples.

The calculated intra-day and inter-day precisions, as percentage relative standard deviation (RSD%), are reported in Table S4 in the Supplementary information. These data (lower than 10% for all the analytes) confirmed the repeatability of the optimized method.

#### Matrix effect

Based on the extraction recovery data obtained, the potential presence of a matrix effect was investigated to identify the influence of sludge on the analytical results, by examining the surrogate compounds and comparing the slopes of two sets of calibration lines (Fig. S2, Table S3): one set from standard solutions in solvent and the other from matrix-matched calibrations. The comparison of the slopes was performed using a Student’s *t*-test, as reported in the “Figures of merit” section and detailed in paragraph S3 of the Supplementary information.

The observation of lower slopes in the matrix-matched curves, as compared to those in a solvent solution, suggests the occurrence of ion suppression in the GC–MS analysis due to the sludge matrix. This finding could indicate that the matrix components might be impacting the ionization efficiency of the analytes in the mass spectrometer, leading to a decrease in the response for the same concentration of analytes in the matrix-matched samples compared to the solvent solution samples. However, according to the comparison between *t*_calc_ and *t*_tab_ (2.03 for all compounds, with 20 + 20 – 4 = 36 degrees of freedom), this ME could be considered negligible.

**Fig. 2:**
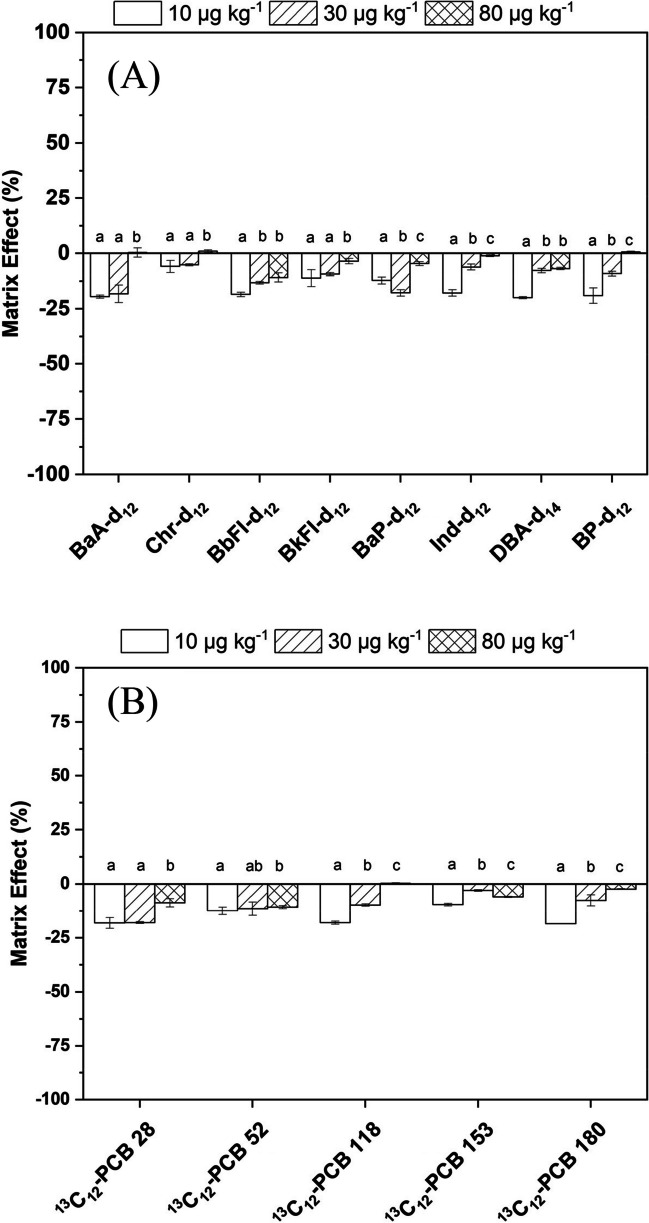
Mean values (*n* = 3) of matrix effects obtained for target PAHs (**A**) and PCBs (**B**) surrogates through the optimized protocol, at three different spiking levels: 10 µg kg^−1^, 30 µg kg^−1^, and 80 µg kg^−1^. Error bars refer to standard deviation. Bars labeled with different letters pointed out statistically different mean values (Dunnett T3 nonparametric contrast test, *p* ≤ 0.05). Conversely, bars with at least one letter in common represent mean values that are not statistically different from each other

Punctual MEs, represented in Fig. 2A, B, evaluated by Eq. 2, at concentration levels including the MDL, confirmed the results obtained through the statistic treatment with MEs below the maximum accepted values of 20% for all the three concentration levels tested. The absence of signal enhancement in the matrix agrees with the effectiveness of the chromatographic separations. Furthermore, it was observed that, for the majority of the analytes, punctual ME values are statistically different across the concentration levels (Dunnet T3 contrast test) and in, particular, that the extent of suppressive effects, which can impact the detection and quantification of PAHs and PCBs, diminishes as the concentration of these compounds increases. This trend indicates that at higher concentrations of PAHs and PCBs, the influence of the matrix on the analytical signal is less pronounced, leading to more accurate and reliable measurement outcomes. Typical chromatograms obtained for the analysis of a sludge matrix, a sludge matrix spiked with standards, and a cyclohexane mixture of the standard are reported in Fig. S3 in the Supplementary information.

The comparison of the matrix effect within the method developed and those present in the literature is not straightforward since to the best of our knowledge, this parameter was never investigated in the determination of PAHs or PCBs in sludge.

### Method greenness

For the method developed, in addition to the figures of merit (“Method validation” section), its greenness was also evaluated. For this purpose, the AGREE software proposed in 2020 by Pena-Pereira et al. (2020) was used, which considers the twelve principles of green chemistry converting them into scores between 0 (the least green alternative) and 1 (the greenest alternative). The final output is a clock-like graph in a green-yellow–red color scale showing the overall weighted score.

**Fig. 3:**
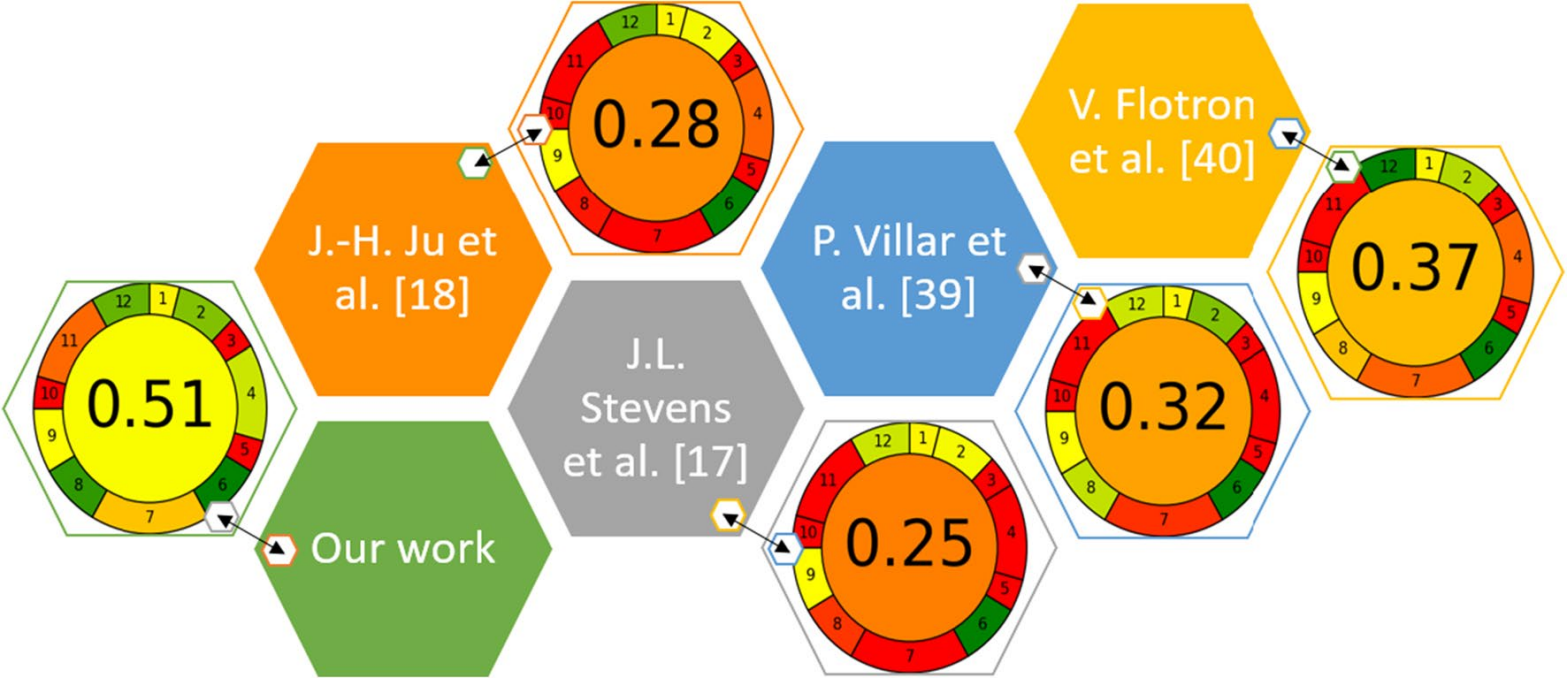
Greenness of the method here developed in comparison with other studies dealing on micropollutant determination in sewage sludge. Assessment is performed according to the green chemistry analysis (weight—*w*—of each criteria is indicated in parenthesis) 1: sample treatment (w 1); 2: sample amount (w 2); 3: device positioning (w 1); 4: number of preparation steps (w 3); 5: automation/miniaturization (w 1); 6: derivatization (w 2); 7: waste generated (w 4); 8: number of analytes determined (w 2); 9: energy consumption (w 2); 10: source of reagents (w 1); 11: toxicity of the reagents (w 3); 12: safety of the operator (w 2)

For comparison, the method here developed and four procedures for determination of PAHs or PCBs in sewage sludge samples (Flotron et al. 2003; Ju et al. 2009; Stevens et al. 2003; Villar et al. 2004) were evaluated with AGREE (Fig. 3). Briefly, the methods considered were based on Soxhlet extraction with GC–MS analysis (Ju et al. 2009; Stevens et al. 2003) and microwave extraction with HPLC–DAD or fluorescence (Flotron et al. 2003; Villar et al. 2004). According to the AGREE procedure, the method developed here obtained a final score of 0.51 with respect to the other methods which obtained a final score included within 0.25 and 0.37. In more detail, the method presented here provides significant improvements in criteria 2 (lower amount of sample processed) (Flotron et al. 2003; Ju et al. 2009; Stevens et al. 2003), 4 (lower number of operational steps, e.g., solvent change not necessary, fewer clean-up steps) (Flotron et al. 2003; Ju et al. 2009; Stevens et al. 2003; Villar et al. 2004), 7 (lower volume of solvent used, and hence lower volumes of waste generated) (Flotron et al. 2003; Ju et al. 2009; Stevens et al. 2003; Villar et al. 2004), 8 (high number of analytes determined per analytical run, i.e., throughput) (Flotron et al. 2003; Ju et al. 2009; Stevens et al. 2003; Villar et al. 2004), and 11 (toxicity of the solvents used for extraction/clean-up) (Flotron et al. 2003; Ju et al. 2009; Stevens et al. 2003; Villar et al. 2004).

### Sludge analysis

**Fig. 4:**
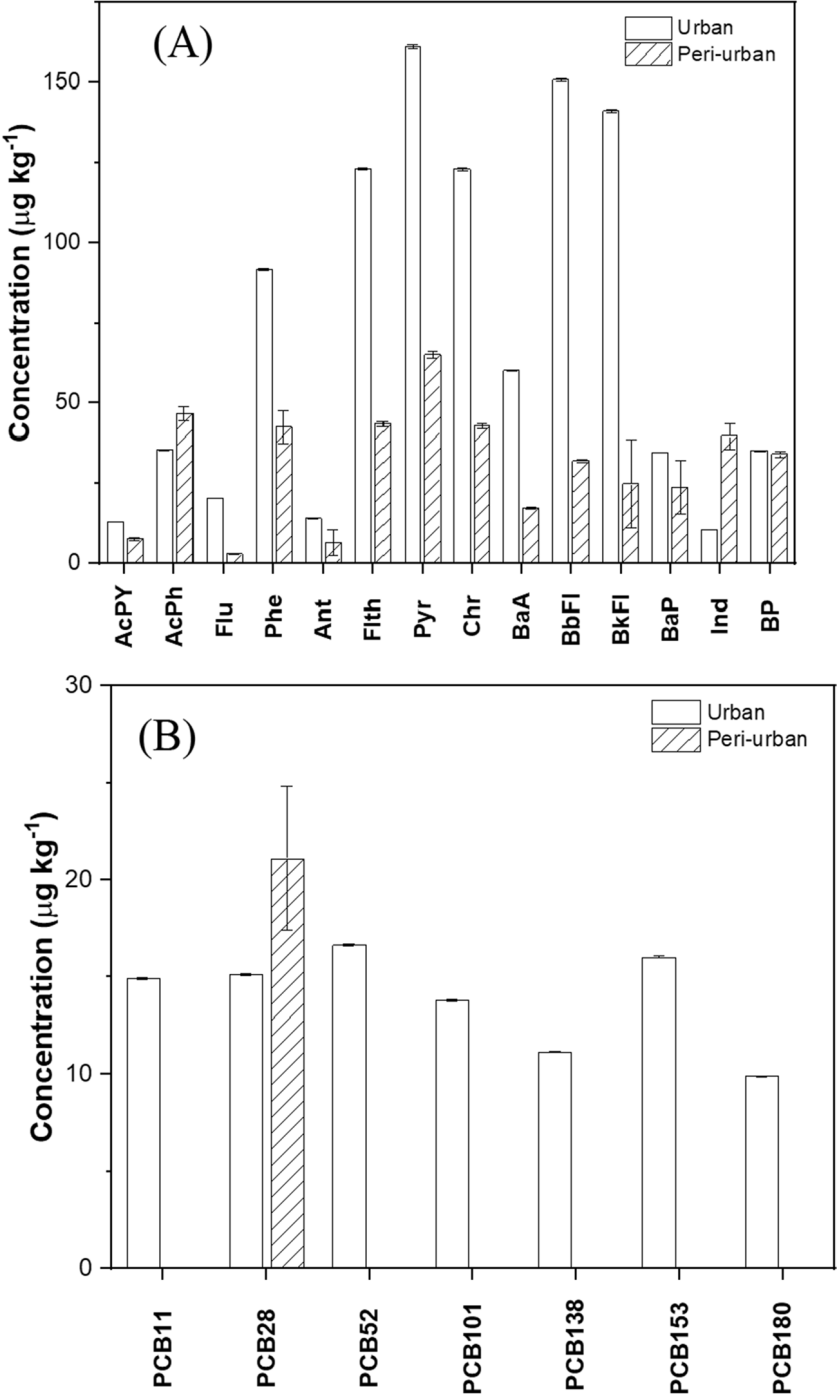
Occurrence of PAHs (**A**) and PCBs (**B**) in digested sludges from wastewater treatment plants located in urban and rural areas. Analysis was performed with the optimized method experimental test no. 5). Mean concentrations and standard deviation bars (*n* = 3 replicates) are reported

The optimized procedure (“Final extraction and purification conditions” section) was used to analyze PAHs and PCBs in two digested sludges derived from urban (sludge #1) and peri-urban (sludge #2) areas characterized by a different industrial impact. The samples were analyzed in triplicates, together with two blanks to exclude laboratory contamination. Results are reported in Fig. 4 and Table 2.

**Table 2:** Concentrations of PAHs in sludges and predicted concentrations (PEC, µg kg^−1^) in sludge-amended soils. Environmental risk data, i.e., predicted no-effect concentration (PNEC, µg kg^−1^), and risk quotients (*RQ*) are also reported for each PAH compound

PAHs	*C*_sludge#1_ (µg kg^−1^)	*C*_sludge#2_ (µg kg^−1^)	PEC soil#1	PEC soil#2	PNEC soil^a)^	RQsoil#1	RQsoil#2
Naphthalene	n.d	n.d	-	-	176	-	-
Acenaphthylene	12.8	7.4	0.0188	0.0109	5.87	0.00321	0.00185
Acenaphthene	35.5	46.6	0.0522	0.0685	6.71	0.00778	0.01021
Fluorene	20.3	n.d	0.0298	-	77.4	0.00039	-
Phenanthrene	91.9	42.4	0.1351	0.0623	204	0.00066	0.00031
Anthracene	13.9	6.3	0.0204	0.0093	57.2	0.00036	0.00016
Fluoranthene	123.1	43.4	0.1810	0.0638	423	0.00043	0.00015
Pyrene	161.3	65.1	0.2372	0.0957	195	0.00122	0.00049
Benzo[a]anthracene^b^	60.0	17.1	0.0882	0.0251	108	0.00082	0.00023
Chrysene^b^	123.0	42.7	0.1809	0.0628	166	0.00109	0.00038
Benzo[b]fluoranthene^b^	150.8	31.9	0.2218	0.0469	n.a	-	-
Benzo[k]fluoranthene^b^	141.3	24.8	0.2078	0.0365	240	0.00087	0.00015
Benzo[a]pyrene^b^	34.5	23.8	0.0507	0.0350	150	0.00034	0.00023
Indeno[1.2.3-cd]pyrene^b^	10.3	39.6	0.0151	0.0582	33	0.00046	0.00176
Dibenz[a.h]anthracene^b^	n.d	n.d	-	-	170	-	-
Benzo[ghi]perylene	35.1	34.0	0.0516	0.0500	200	0.00026	0.00025
Σ PAHs	1013.8	425.1	1.491	0.625		0.0179	0.162
Σ LPAHs^c^	174.4	102.7					
Σ HPAHs^d^	839.4	322.4					
Σ CPAHs^b^	519.9	179.9					

*n.d.*: not detected in sludge, *n.a.* not available

^a^PNEC values were taken fromSun et al. (2019)

^b^Carcinogenic PAHs (CPAHs) according to US-EPA (1993)

^c^Sum of 2- and 3-ring PAHs (naphthalene, acenaphthylene, acenaphthene, fluorene, phenanthrene, anthracene)

^d^Sum of 4-, 5-, and 6-ring PAHs (fluoranthene, pyrene, benzo[a]anthracene, chrysene, benzo[b]fluoranthene, benzo[k]fluoranthene, benzo[a]pyrene, indeno[1.2.3-cd]pyrene, dibenz[a.h]anthracene, and benzo[ghi]perylene)

As regards sludge #1, all PAHs were detected except naphthalene (Naph) and dibenz[a,h]anthracene (DBA) which were below the MDL values. The maximum contamination is accounted for by pyrene (Pyr). The sum of the nine regulated PAHs is 716 µg kg^−1^, well below the limit set. The sum of the analyzed PCBs was 97 µg kg^−1^, with PCB 15 and dioxin-like PCB 81, PCB 118, PCB 123, PCB 167, PCB 169, and PCB 189 below the MDL limit. Also for PCBs, the regulated threshold is fully respected.

Even for sludge #2, many PAH compounds were detected, and Pyr is the PAH contaminant present at higher concentrations, whereas naphthalene (Naph), fluorene (Flu), and dibenz[a,h]anthracene (DBA) are below the MQL values. In sludge derived from peri-urban area, the sum of the nine regulated PAHs is 279 µg kg^−1^, and the only PCB detected is PCB 28.

Overall, the contamination of sludge derived from wastewater plant located in urban/industrialized area is higher than the one observed for sludge derived from the wastewater plant located in peri-urban area. Analyzing these data in more detail, we observe that the low-molecular-weight PAH (ΣLPAHs, the sum of 2- and 3-ring PAHs: naphthalene, acenaphthylene, acenaphthene, fluorene, phenanthrene, anthracene) account for 174.1 µg kg^−1^ (17% of total PAHs) and 102.7 µg kg^−1^ (24% of total PAHs) for sludge #1 and sludge #2, respectively, indicating a quite similar contribution of LPAHs in the contamination, regardless the sludge provenience. On the contrary, the greater difference in contamination between sludge #1 and sludge #2 is explained by the content of high-molecular-weight PAH (ΣHPAHs, sum of 4-, 5- and 6-ring PAHs: fluoranthene, pyrene, benzo[a]anthracene, chrysene, benzo[b]fluoranthene, benzo[k]fluoranthene, benzo[a]pyrene, indeno[1.2.3-cd]pyrene, dibenz[a.h]anthracene, benzo[ghi]perylene) which are much more abundant in urban sludge (839.4 µg kg^−1^) than in peri-urban sludge (322.4 µg kg^−1^). Within this class, the 4-ring compounds are predominant accounting for 46% (sludge #1) and 40% (sludge #2) of the total sixteen PAHs (Σ PAHs), followed by the 5-ring compounds accounting for 33% (#1) and 12% (#2) of Σ PAHs. This distribution is in good agreement with the one observed by Chen et al. (2019) and references herein, which noted the predominance of pyrene and fluoranthene, as in our case. A better understanding of the major occurrence of these compounds in sludge is not easy. Differently from what is usually done in many environmental studies (e.g., on sediments and water), the source of PAHs (e.g., petrogenic or pyrogenic; fuel or wood combustion) with the aid of various molecular diagnostic ratios was not attempted, in agreement with the considerations elucidated by Katsoyiannis et al. (2007). In fact, the condition that the ratios remain constant *en route* is not satisfied due to (*i*) the mixing and homogenization of wastewaters that takes place during the transportation; (*ii*) the treatment that the wastewaters undergo at the various treatment steps in which each pollutant behaves differently according to its intrinsic physico-chemical characteristics (sorption on the solids in the primary sedimentation step; sorption in the suspended solids, i.e., the partition between water and sludge; volatilization, air stripping, biotransformation).

According to the latest available data of contamination for sewage sludge derived from urban wastewater collected across the Northern part of Italy (Suciu et al. 2015), total PAH content (Σ PAHs) determined here, i.e., 279 and 176 µg kg^−1^, is lower than the maximum content monitored in 2013 (1171 µg kg^−1^) and in 2015 (3917 µg kg^−1^). The Σ PAHs determined here are lower than the ones reported for urban plants monitored in Taiwan, Tunisia, UK, Spain, China, Switzerland, Korea, and Poland but higher than those reported for Jordan case studies (around 35 µg kg^−1^) (Chen et al. 2019).

As regards the PAHs defined as carcinogenic according to EPA (United States Environmental Protection Agency (US-EPA) (1993)), they are present at 520 µg kg^−1^ (sludge #1) and 180 µg kg^−1^ (sludge #2) and account for 51% and 42% of the total PAHs contamination level. A risk analysis (“Risk assessment for sludge application” section) could give further indications and information on sludge re-use.

### Risk assessment for sludge application

Since carcinogenic PAHs according to EPA were detected in the sludges derived from the treatment of wastewater in urban and peri-urban districts, even if fully compliant with the EU-regulated values, sludges were additionally studied for possible application in soil. In detail, we investigated the environmental risk (“Predicted concentrations in soil and environmental risk assessment due to PAHs in sludge-amended soil” section) and the carcinogenic human risk through dermal contact (“Risk for human exposure through dermal contact” section) caused by the spread of the sludge. The risk assessment was performed for PAHs and not for PCBs, due to the lower availability of ecological data on this last class of compounds.

#### Predicted concentrations in soil and environmental risk assessment due to PAHs in sludge-amended soil

The predicted concentrations (PEC) in sludge-amended soil (1 year of application) were calculated according to Eq. 3, using the concentrations of PAHs measured in sludges through the optimized method. For each of the PAH compounds analyzed here, the predicted concentration in both soils (see Table 2) is well below the limits (ranging from 100 to 5000 µg kg^−1^) established by Italian National Regulation 152/2006 for public green areas which are assimilated to agricultural lands. Risk quotients (RQ), also shown in Table 2, calculated through the PEC and PNEC values using Eq. 4, are below 0.1, indicating that the environmental risk in soils when the two sludges had been applied after 1 year is low (Hernando et al. 2006).

#### Risk for human exposure through dermal contact

The carcinogenic risk through dermal contact with sludge-amended soil was assessed within an adult gardener exposure scenario. BaP is assumed as the model compound for the PAH mixture, thus, all concentrations of PAH compounds were expressed by BaP_eq_ which was calculated based on the TEFs reported by Gungormus et al. (2014). The sum of PAHs expressed as BaP_eq_ was 0.000107 mg kg^−1^ for soil amended with sludge #1 and 0.0000532 mg kg^−1^ for soil amended with sludge #2.

The lifetime average daily dose from dermal contact (LADD) was calculated according to Eq. 5 using the parameters reported in Table S5 of the Supplementary information section and resulted to be 2.32·10^−11^ mg kg^−1^ d^−1^ for soil amended with sludge #1 and 1.15·10^−11^ mg kg^−1^ d^−1^ for soil amended with sludge #2. Subsequently, the carcinogenic risk (*R*) was calculated by Eq. 6 using oral slope factor of 1 (mg kg^−1^ d^−1^) ^−1^ (United States Environmental Protection Agency (US-EPA) (2013)), 12 and 25 (mg kg^−1^ d^−1^) ^−1^ as proposed by Gungormos (2014) and by Knafla et al. (2006), respectively. The higher carcinogenic risk was 3.3·10^−10^ for soil amended with sludge #1 and 1.6·10^−10^ for soil amended with sludge #2. Considering that exposure to carcinogenic compounds derives from many sources, like dietary and inhalation exposure (not accounted in this work), and that the acceptable cumulative carcinogenic risk level is 10^−6^, the dermal exposure to these kinds of sludge-amended soils represents a negligible exposure factor.

## Conclusions

Sludge derived from wastewater treatment represents a social and environmental issue, both for its management and for the risks associated with its valorization within the circular economy policies for the possible residual contamination. In this regard, a validated analytical protocol for the assessment of sludge contamination is mandatory for an accurate risk analysis.

For the first time, this research has provided, through chemometric optimization, a reliable green method for the simultaneous determination of PAHs and PCBs in anaerobically digested sludges derived from wastewater treatment plants based on microwave extraction, a simple extract clean-up, and GC–MS analysis. This method allows one to overcome the current lack of literature since, overall, less than 20 mL is required for the extraction and cleaning of the extract and less than 2 h for the preparation and simultaneous analysis of PAHs and PCBs.

The method, optimized in sludge deriving from urban district, provided detection limits (below 11.5 µg kg^−1^ for PAHs and 13.7 µg kg^−1^ for PCBs) below the limits fixed by EC and below the average sludge contamination reported in the literature. For PAHs only, a comparison with literature studies was possible, noting that the detection limits are even lower than those obtained through methods using longer and more complex extraction and clean-up procedures with preconcentration. The matrix effect, which is not generally studied in the literature reviewed, evaluated here by a *t*-test and by the addition of known amounts of pollutants at three concentration levels (including the one corresponding to the method detection limit), indicated a negligible effect (< 20%). The analysis of PAHs and PCBs in digested sludges, representative of scenarios of different anthropic impact, indicated that the amount and the number of PAHs and PCBs detected were higher in the sludge derived from the urban district and mainly represented by the 4-ring PAHs, especially pyrene and fluoranthene, in line with available data of sludge contamination in Italy. Six out of the seven compounds classified as carcinogenic by EPA were detected, accounting for about 50% of total PAHs; the concentrations detected for both classes of pollutants were anyway well below the current regulations. The concentrations detected in both sludges, treated to simulate a possible land application scenario, indicated a low environmental risk for soil and a low risk for human exposure through dermal contact but also highlighted the importance of quantifying the human health risk in sludge management.

## Supplementary Information

Below is the link to the electronic supplementary material.

Supplementary file1 (DOCX 376 KB)

## Data Availability

The authors declare that the data supporting the findings of this study are available within the paper and its Supplementary Information files. Should any raw data files be needed in another format, they are available from the corresponding author upon reasonable request.
