# Supplementary material for: Determination of persistent organic pollutants in urban and peri-urban wastewater sludge: environmental and carcinogenic human risk assessment in the case of land application
Source: Environ Sci Pollut Res Int. 2024 Jul 22;33(22):11148–61. doi: 10.1007/s11356-024-34420-5 (PMC13415280; doi:10.1007/s11356-024-34420-5)
Supplement: Supplementary file 1 — Supplementary file1 (DOCX 376 KB) [file 11356_2024_34420_MOESM1_ESM.docx]

### SUPPLEMENTARY MATERIAL

**Determination of persistent organic pollutants in urban and peri-urban wastewater sludge: environmental and carcinogenic human risk assessment in the case of land application**

Maria Concetta Bruzzoniti*^a^, Vander Tumiatti^b^, Armando Quazzo^c^, Mihail Simion Beldean-Galea^d^,

Massimo Del Bubba^e^, Luca Rivoira*^a^

^a^ Department of Chemistry, University of Turin, Via P. Giuria 5, 10125, Turin, Italy

^b^ Sea Marconi Technologies, Via Ungheria 20, 10093, Collegno, Italy

^c^ Società Metropolitana Acque Torino, SMAT, S.p.A. Corso XI Febbraio 22, 10152 Turin, Italy

^d^ Babes-Bolyai University, Faculty of Environmental Science and Engineering, Cluj-Napoca, Romania.

^e^ Department of Chemistry “Ugo Schiff”, University of Florence, Via della Lastruccia 3, Sesto Fiorentino, 50019, Italy

*S1. Chromatographic analysis*

For PAHs and PCBs analysis, an Agilent 6980 series gas chromatograph (with Agilent 7683 Series autosampler) and an Agilent 5973 Network MS detector controlled by Agilent ChemStation software were used. The GC column was a (5%-Phenyl)-methylpolysiloxane column (DB-5 ms, 30 m × 0.25 mm × 25 μm; Agilent). Helium was used as gas carrier (1 mL min^-1^). MS detection was performed in Single Ion Monitoring (SIM) mode at proper m/z ratio (see Tables S1 and S2). Injections (2 μL) were performed by the pulsed splitless mode (pressure at 40 psi for 2 minutes). The oven ramp was set as follows: starting temperature: 40 °C (80 °C for dichloromethane), hold for 2 min; ramp to 176 °C, 12°C min^-1^ rate; ramp to 196 °C, 5 °C min^-1^ rate, hold for 3 mins; ramp to 224 °C, 12°C min^-1^ rate; ramp to 244 °C, 12 °C min^-1^ rate, hold for 3 min; ramp to 270 °C, 7 °C min^-1^ rate, hold for 3 min; final ramp to 300 °C, 5 °C min^-1^, hold for 10 min to completely clean and restore the GC column. These conditions allowed to achieve complete separation of the 16 PAHs and 14 PCBs within 49 min.

S2. Calculation of figure of merits of the optimized protocol

Method detection limits (MDL) and method quantitation limits (MQL) for the 30 target compounds were calculated by means of the response error and the slope of the calibration curve, according to the expression MDL= 3.3 Sy/m, and MQL= 10 Sy/m, where Sy = response error; m = slope of the calibration (Shrivastava and Gupta, 2011).

*S3. Statistical approach for matrix effect evaluation*

To choose the correct statistic approach, the residual variances of both curves were compared through an F-test for each surrogate. In detail, if the residual variances could be considered similar ($F_{calc}<F_{tab,\alpha,n_{1}-1,n_{2}-1}$ with α=0.05, and n_1_ and n_2_ = number of calibration levels in solvent and matrix-matched curve, respectively), the Student’s t value should be calculated as follows:

$t_{calc}=\frac{b_{1}-b_{2}}{\sqrt{s_{ep}^{2}\left( \frac{1}{\sum\left( x_{i1}-\bar{x}_{1} \right)^{2}}+\frac{1}{\sum\left( x_{i2}-\bar{x}_{2} \right)^{2}} \right)}}$ (S1)

with the pooled estimates variance $s_{ep}^{2}$calculated as:

$s_{ep}^{2}=\frac{\left( n_{1}-2 \right)*s_{e_{1}}^{2}+\left( n_{2}-2 \right)*s_{e_{2}}^{2}}{n_{1}+n_{2}-4}$ (S2)

where *b* is the intercept of the equation curve, $\sum\left( x_{i1}-\bar{x}_{1} \right)^{2}$ the summatory of the concentration levels for each curve, *n* is the number of calibration levels (in this case 20 for each curve), and S is the standard deviation of the slope.

Data treatment can indicate that there is no significant difference between the slopes of both calibration curves (with a probability of 95%), meaning that no matrix effect is present, when the t_calc_ is below the t_tab_ extracted from a t-table, with n_1_ + n_2_ −4 degrees of freedom.

Conversely, if the residual variances are not similar ($F_{calc}>F_{tab,\alpha,n_{1}-1,n_{2}-2}$) the theoretical t` value, to be compared with t_calc_ (calculated as in Equation S1), should be calculated as follows:

$t^{'}=\frac{t_{1}*s_{b_{1}}^{2}+t_{2}*s_{b_{2}}^{2}}{s_{b_{1}}^{2}+s_{b_{2}}^{2}}$ (S3)

where $s_{b_{1}}^{2}=\frac{s_{e_{1}}^{2}}{\sum\left( x_{i1}-\bar{x}_{1} \right)^{2}}$, $s_{b_{2}}^{2}=\frac{s_{e_{2}}^{2}}{\sum\left( x_{i2}-\bar{x}_{2} \right)^{2}}$ and t_1_ and t_2_ are the values obtained from a t-table at the chosen level of significance and n_1_-2 and n_2_-2 degrees of freedom, respectively.

**Table S1** – List of PAH and PCB compounds and surrogates studied throughout this work and m/z values used (in parenthesis).

| **PAHs** | **Acronym (m/z)** | **SIM m/z quantitative ratios** | | **PCBs** | **Acronym** | **SIM m/z quantitative ratios** |  |
| --- | --- | --- | --- | --- | --- | --- | --- |
| naphthalene | Naph | 128 | | 3,3′-dichlorobiphenyl | PCB 11 | 222 |  |
| acenaphthylene | AcPY | 152 | | 4,4′-dichlorobiphenyl | PCB 15 | 222 |  |
| acenaphthene | AcPh | 152 | | 2,4,4′-trichlorobiphenyl | PCB 28 | 186 |  |
| fluorene | Flu | 166 | | 2,2′,5,5′-tetrachlorobiphenyl | PCB 52 | 292 |  |
| phenanthrene | Phe | 178 | | 3,4,4′,5-tetrachlorobiphenyl | *PCB 81 | 292 |  |
| anthracene | Ant | 178 | | 2,2′,4,5,5′-pentachlorobiphenyl | PCB 101 | 254 |  |
| fluoranthene | Flth | 202 | | 2,3′,4,4′,5-pentachlorobiphenyl | *PCB 118 | 326 |  |
| pyrene | Pyr | 202 | | 2′,3,4,4′, 5-penta- chlorobiphenyl | *PCB 123 (326) | 326 |  |
| benzo[a]anthracene | BaA | 228 | | 2,2′,3,4,4′,5-hexachlorobiphenyl | PCB 138 | 360 |  |
| chrysene | Chr | 228 | | 2,2′,4, 4′,5,5′-  hexachlorobiphenyl | PCB 153 | 360 |  |
| benzo[b]fluoranthene | BbFl | 252 | | 2,3′,4,4′,5, 5′-  hexachlorobiphenyl | *PCB 167 | 360 |  |
| benzo[k]fluoranthene | BkFl | 252 | | 3,3′,4,4′,5,5′-  hexachlorobiphenyl | *PCB 169 | 360 |  |
| benzo[a]pyrene | BaP | 252 | | 2,2′,3,4,4′,5,5′-  heptachlorobiphenyl | PCB 180 | 394 |  |
| indeno[1,2,3- cd]pyrene | Ind | 276 | | 2,3,3′,4,4′,5,5′-  heptachlorobiphenyl | *PCB 189 | 394 |  |
| dibenz[a,h]anthracene | DBA | 278 | |  |  |  |  |
| benzo[ghi]perylene | BP | 276 | |  |  |  |  |
| chrysene-d12 | Chr-d12 | 240 | | 2,2′,5,5′-tetrachlorobiphenyl-  ^13^C12 | ^13^C12-PCB 52 | 304 |  |
| benzo[b]fluoranthene-d12 | BbFl-d12 | 264 | | 2,3′,4,4′,5-  pentachlorobiphenyl-^13^C12 | ^13^C12-PCB 118 | 338 |  |
| benzo[k]fluoranthene-d12 | BkFl-d12 | 264 | | 2,2′,4, 4′,5,5′-  hexachlorobiphenyl-^13^C12 | ^13^C12-PCB 153 | 372 |  |
| benzo[a]pyrene-d12 | BaP-d12 | 264 | | 2,2′,3,4,4′,5,5′-  heptachlorobiphenyl-^13^C12 | ^13^C12-PCB 180 | 406 |  |
| indeno[1,2,3-cd]pyrene-  d12 | Ind-d12 | 288 | |  |  |  |  |
| dibenz[a,h]anthracene-d14 | DBA-d14 | 292 | |  |  |  |  |
| benzo[ghi]perylene d12 | BP-d12 | 288 | |  |  |  |  |
|  |  | | Internal Standards | | | | |
| Anthracene-d10 | A-d10 | 188 | | 2,3′,4′,5- tetrachlorobiphenyl-  ^13^C12 | ^13^C12-PCB 70 | 304 |  |

^*^ Dioxin-like PCBs.

**Table S2** – Method detection and method quantitation limits determined in sludge matrix for the analysis of 16 PAHs and the 14 PCBs (*: dioxin-like congeners).

| PAHs | MDL (µg kg^-1^) | MQL (µg kg^-1^) | PCBs | MDL (µg kg^-1^) | MQL (µg kg^-1^) |
| --- | --- | --- | --- | --- | --- |
| Naph | 5.9 | 18.0 | PCB 11 | 10.7 | 32.5 |
| AcPY | 5.8 | 17.7 | PCB 15 | 10.1 | 30.7 |
| AcPh | 6.3 | 19.0 | PCB 28 | 10.4 | 31.4 |
| Flu | 6.4 | 19.5 | PCB 52 | 11.1 | 33.7 |
| Phe | 4.6 | 14.1 | *PCB 81 | 8.9 | 27.1 |
| Ant | 5.1 | 15.6 | PCB 101 | 9.3 | 28.1 |
| Flth | 4.8 | 14.6 | *PCB 118 | 7.9 | 23.9 |
| Pyr | 5.9 | 17.9 | *PCB 123 | 12.5 | 37.9 |
| BaA | 8.8 | 26.5 | PCB 138 | 13.7 | 41.6 |
| Chr | 8.4 | 25.6 | PCB 153 | 12.0 | 36.3 |
| BbFl | 7.3 | 22.1 | *PCB 167 | 13.5 | 41.0 |
| BkFl | 8.8 | 26.6 | *PCB 169 | 8.4 | 25.6 |
| BaP | 8.9 | 27.1 | PCB 180 | 7.0 | 21.1 |
| Ind | 11.5 | 34.8 | *PCB 189 | 6.9 | 20.8 |
| DBA | 8.0 | 24.3 |  |  |  |
| BP | 5.0 | 15.1 |  |  |  |

**Table S3 –** Equation curves, correlation coefficients (R^2^) and t_calc_ factors obtained for both solution in solvent and matrix-matched curves in the matrix effect evaluation. The t_tab_ value (36 degrees of freedom, α=0.05) is 2.03.

|  | Standard solution curve | R^2^ standard solution | Matrix-matched curve | R^2^ matrix-matched | t_calc_ |
| --- | --- | --- | --- | --- | --- |
| BaA-d_12_ | y=0.173x-0.04 | 0.991 | y=0.166x-0.07 | 0.996 | 1.54 |
| Chr-d_12_ | y=0.193x-0.04 | 0.992 | y=0.185x-0.10 | 0.991 | 1.49 |
| BbFl-d_12_ | y=0.143x-0.04 | 0.990 | y=0.134x-0.06 | 0.991 | 1.88 |
| BkFl-d_12_ | y=0.185x-0.04 | 0.991 | y=0.177x-0.08 | 0.997 | 1.77 |
| BaP-d_12_ | y=0.156x-0.04 | 0.996 | y=0.161x-0.08 | 0.993 | 1.14 |
| Ind-d_12_ | y=0.086x-0.03 | 0.990 | y=0.086x-0.01 | 0.997 | 0.15 |
| DBA-d_14_ | y=0.072x-0.002 | 0.998 | y=0.069+0.009 | 0.997 | 1.44 |
| BP-d_12_ | y=0.097x+0.09 | 0.989 | y=0.088x-0.01 | 0.992 | 0.47 |
| ^13^C_12_-PCB 28 | y=0.176x-0.03 | 0.998 | y=0.186x-0.02 | 0.993 | 1.81 |
| ^13^C_12_-PCB 52 | y=0.119x-0.02 | 0.989 | y=0.118-0.006 | 0.995 | 0.13 |
| ^13^C_12_-PCB 118 | y=0.151x-0.02 | 0.987 | y=0.149x-0.06 | 0.990 | 0.51 |
| ^13^C_12_-PCB 153 | y=0.109x-0.02 | 0.988 | y=0.104x-0.03 | 0.992 | 1.52 |
| ^13^C_12_-PCB 180 | y=0.075x-0.008 | 0.990 | y=0.077x-0.05 | 0.985 | 0.54 |

**Table S4 –** Intra- and inter-day precision on the analysis of surrogates spiked in sludge.

| Surrogate | Relative standard deviation % (RSD) | |
| --- | --- | --- |
|  | **Intra-day (n = 20)** | **Inter-day (n = 20)** |
| BaA-d_12_ | 5.84 | 7.45 |
| Chr-d_12_ | 4.12 | 6.48 |
| BbFl-d_12_ | 8.12 | 8.64 |
| BkFl-d_12_ | 9.32 | 9.81 |
| BaP-d_12_ | 7.21 | 9.12 |
| Ind-d_12_ | 4.11 | 8.54 |
| DBA-d_14_ | 8.95 | 9.41 |
| BP-d_12_ | 4.21 | 7.12 |
| ^13^C_12_-PCB28 | 6.74 | 8.54 |
| ^13^C_12_-PCB52 | 7.52 | 8.99 |
| ^13^C_12_-PCB118 | 7.11 | 8.54 |
| ^13^C_12_-PCB153 | 6.21 | 7.83 |
| ^13^C_12_-PCB180 | 8.32 | 8.77 |

**Table S5** – Input parameters of the dermal contact exposure model. Data were taken from *United States Environmental Protection Agency (US-EPA). Example exposure scenarios 600R03036, 2004*, except for the number of events and exposure duration.

| **Parameter** | **Value** |
| --- | --- |
| CF (kg mg^-1^) | 1·10^-6^ |
| SA/BW (cm^2^ event^-1^ kg^-1^) | 63.2 |
| AF soil (mg cm-2) | 0.096 |
| EF (event y^-1^) | 100 |
| ED (y) | 40 |
| ABS | 0.13 |
| AT (d) | 25550 |
| Cancer slope factor, CSF (mg kg^-1^ die^-1^)^-1^ | |
| a) | 1 |
| b) | 12 |
| c) | 25 |

^a)^ according to *United States Environmental Protection Agency (US-EPA), 2013, Toxicological*

*Review of Benzo[a]pyrene (CASRN 50‐32‐8). In Support of Summary Information on the Integrated Risk Information System (IRIS).*

^b)^ according to *Gungormus E, Tuncel S, Tecer LH, Sofuoglu SC. Inhalation and dermal exposure to atmospheric polycyclic aromatic hydrocarbons and associated carcinogenic risks in a relatively small city. Ecotoxicology and Environmental Safety 2014; 108: 106-113*.

^c)^ according to *Knafla A, Phillipps KA, Brecher RW, Petrovic S, Richardson M. Development of a dermal cancer slope factor for benzo[a]pyrene. Regulatory Toxicology and Pharmacology 2006; 45: 159-168.*


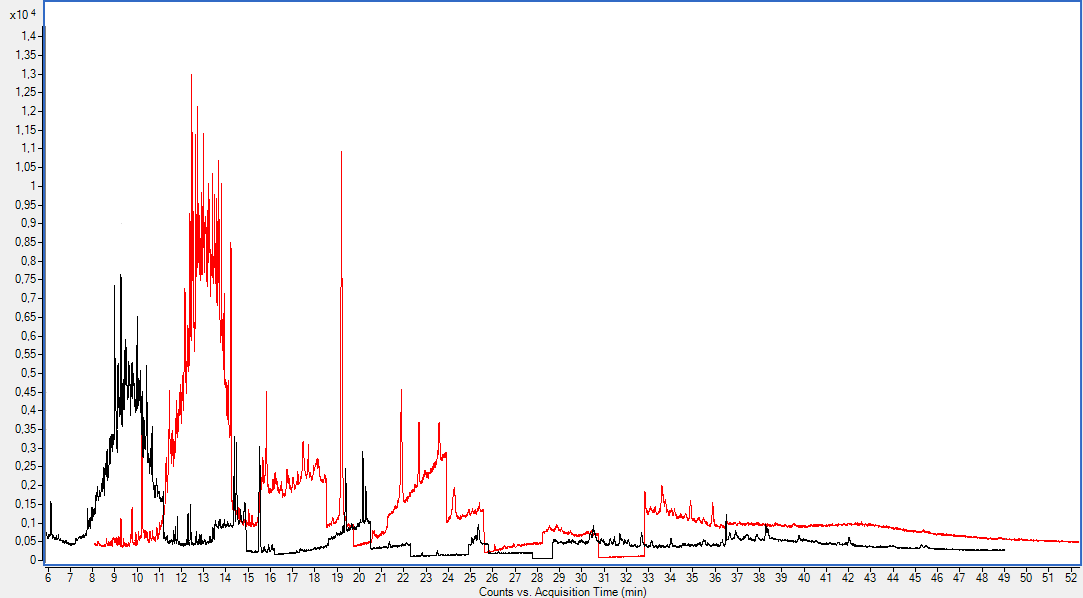


**Figure S1** – Effect of the extracting solvent on the GC background noise. Gas chromatogram tracks (scan mode) sludge #1 samples extracted by dichloromethane, experimental run n. 4 (red track) and cyclohexane, experimental run n. 5 (black track). Instrumental conditions are reported in the manuscript.

**
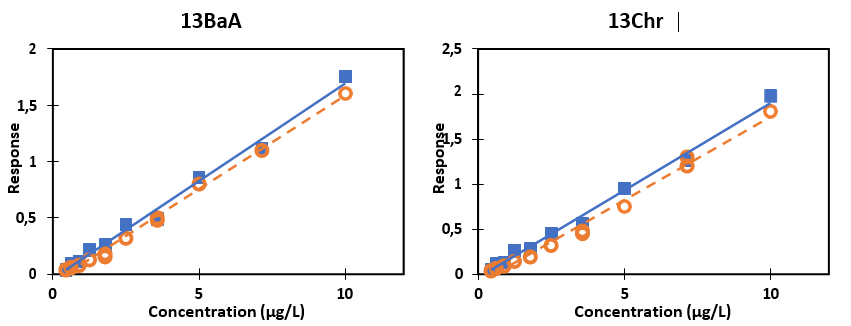

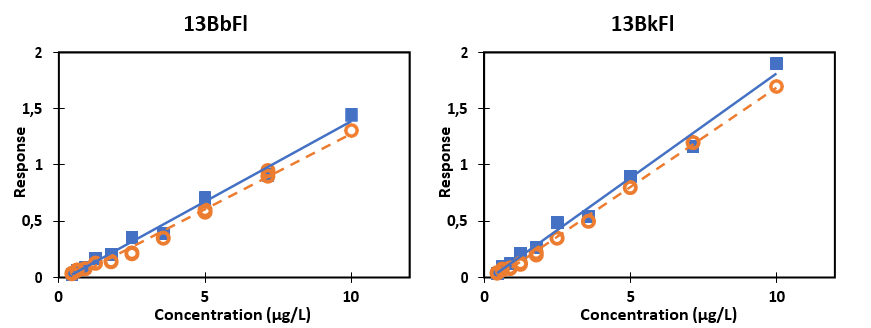

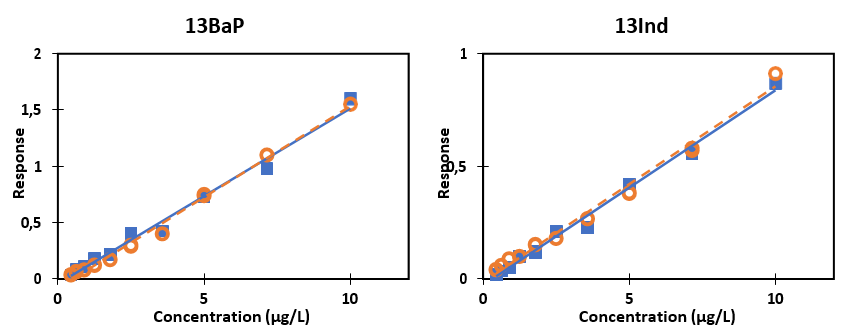

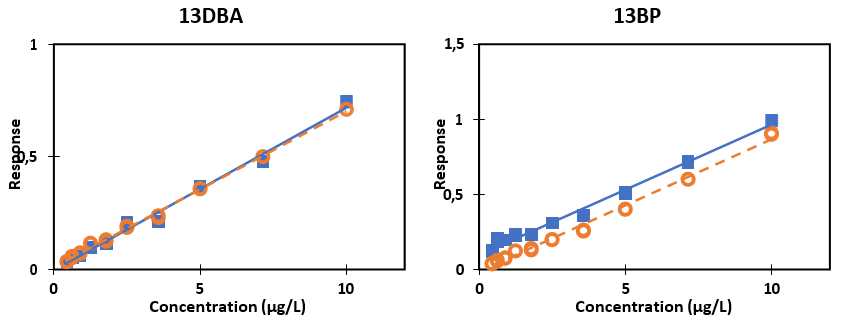
**

**
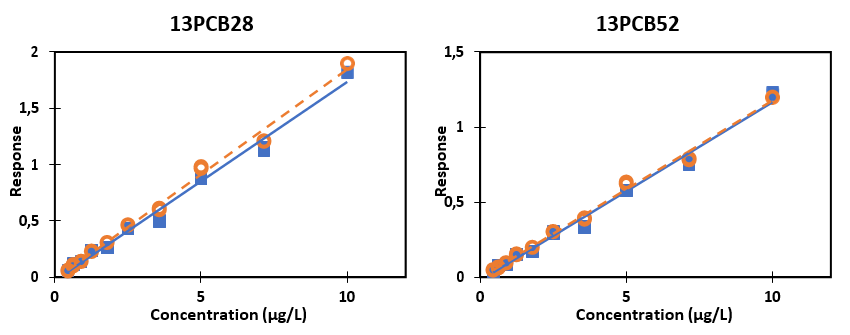

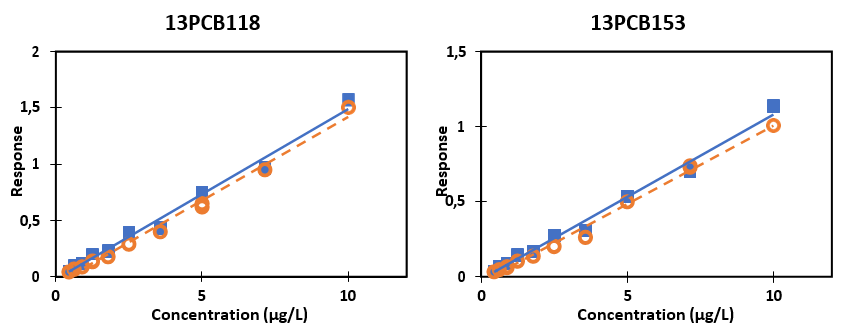

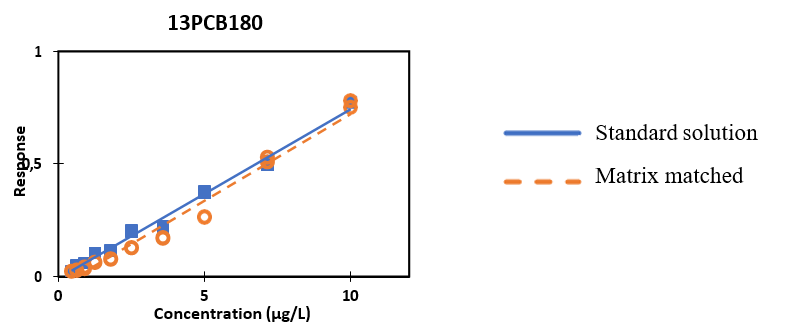
**

**Figure S2 -** Comparison of matrix-matched (dotted orange line) and standard solution (continuous blue line) calibration curves obtained for each surrogate. The response corresponds to the analyte area divided by the corresponding internal standard area.

**
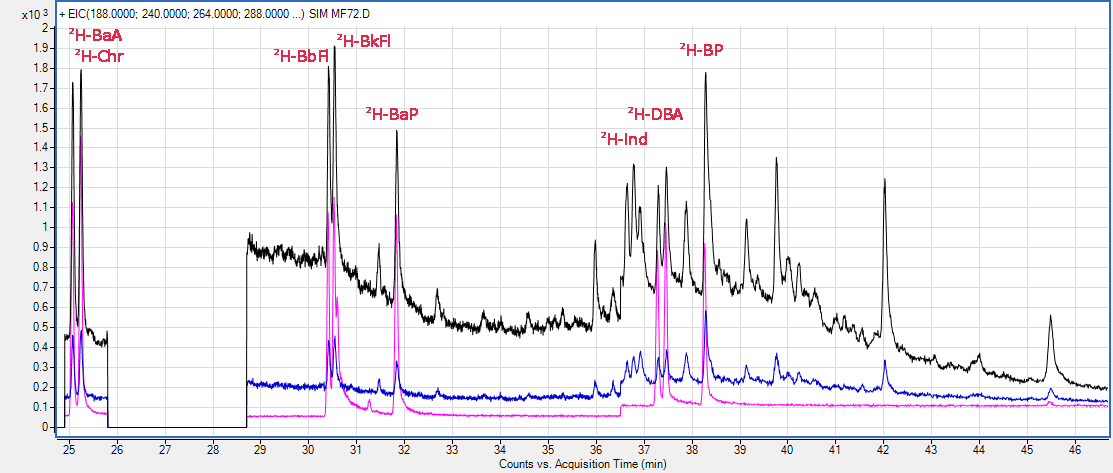
**

**
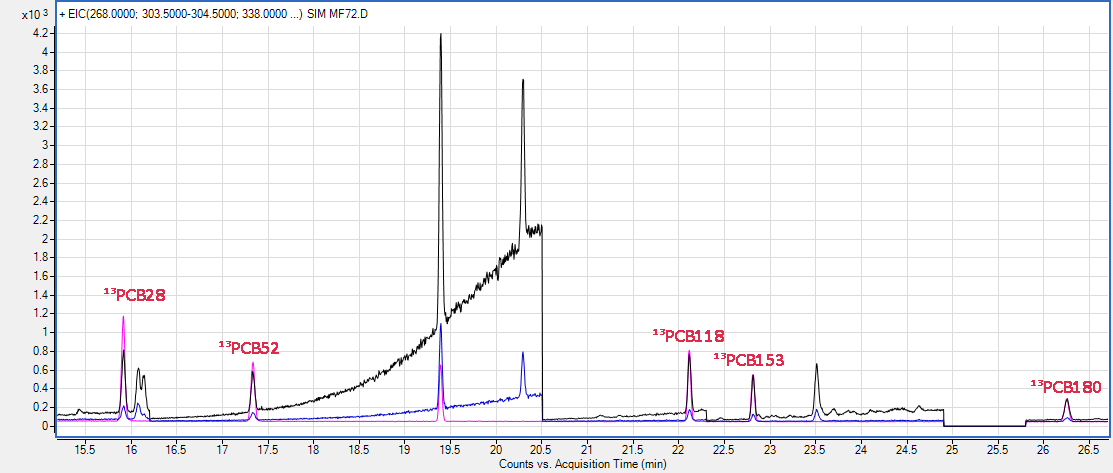
**

**Figure S3** - Overlay of a chromatogram of a sludge matrix (blue), a sludge matrix spiked with standards (black) and cyclohexane mixture of the standard (pink), both at 10 µg/Kg
